# Functional Evolution in Orthologous Cell-encoded RNA-dependent RNA Polymerases*

**DOI:** 10.1074/jbc.M115.685933

**Published:** 2016-02-23

**Authors:** Xinlei Qian, Fursham M. Hamid, Abbas El Sahili, Dina Amallia Darwis, Yee Hwa Wong, Shashi Bhushan, Eugene V. Makeyev, Julien Lescar

**Affiliations:** From the ‡ Division of Structural Biology and Biochemistry, School of Biological Sciences, Nanyang Technological University, 138673 Singapore, Singapore,; the § Medical Research Council Centre for Developmental Neurobiology, King's College, London SE1 1UL, United Kingdom, and; ¶ UPMC UMRS CR7-CNRS ERL 8255-INSERM U1135 Centre d' Immunologie et des Maladies Infectieuses, Faculté de Médecine Pierre et Marie Curie, Centre Hospitalier Universitaire Pitié-Salpêtrière, 75031 Paris, France

**Keywords:** electron microscopy (EM), protein evolution, RNA interference (RNAi), RNA polymerase, x-ray crystallography, RNA-dependent RNA polymerase

## Abstract

Many eukaryotic organisms encode more than one RNA-dependent RNA polymerase (RdRP) that probably emerged as a result of gene duplication. Such RdRP paralogs often participate in distinct RNA silencing pathways and show characteristic repertoires of enzymatic activities *in vitro*. However, to what extent members of individual paralogous groups can undergo functional changes during speciation remains an open question. We show that orthologs of QDE-1, an RdRP component of the quelling pathway in *Neurospora crassa*, have rapidly diverged in evolution at the amino acid sequence level. Analyses of purified QDE-1 polymerases from *N. crassa* (QDE-1*^Ncr^*) and related fungi, *Thielavia terrestris* (QDE-1*^Tte^*) and *Myceliophthora thermophila* (QDE-1*^Mth^*), show that all three enzymes can synthesize RNA, but the precise modes of their action differ considerably. Unlike their QDE-1*^Ncr^* counterpart favoring processive RNA synthesis, QDE-1*^Tte^* and QDE-1*^Mth^* produce predominantly short RNA copies via primer-independent initiation. Surprisingly, a 3.19 Å resolution crystal structure of QDE-1*^Tte^* reveals a quasisymmetric dimer similar to QDE-1*^Ncr^*. Further electron microscopy analyses confirm that QDE-1*^Tte^* occurs as a dimer in solution and retains this status upon interaction with a template. We conclude that divergence of orthologous RdRPs can result in functional innovation while retaining overall protein fold and quaternary structure.

## Introduction

Eukaryotic cells widely use small RNA (sRNA)^4^ guides to limit proliferation of viruses and transposable elements, maintain proper chromosomal structure, and control endogenous gene expression in a sequence-specific manner (1–10). sRNA pathways typically require RNase III-like and PIWI/PAZ proteins that process completely or partially double-stranded RNA (dsRNA) precursors and recruit sRNA products of this reaction into functional silencing complexes (11–13). In fungi, plants, protozoans, and some metazoans, sRNA production often depends on template-dependent RNA synthesis catalyzed by cell-encoded RNA-dependent RNA polymerases (RdRPs) (14, 15).

Many RdRPs contribute to maintenance and amplification of gene silencing signals initiated by primary sRNAs originating from viral genomes, endogenous sequences, or dsRNAs experimentally delivered into a cell (16–23). In such cases, long single-stranded RNA (ssRNA) targets of an initial round of silencing become templates for RdRP-catalyzed RNA synthesis, ultimately giving rise to secondary sRNAs. Moreover, some RdRPs may trigger a silencing response with no apparent need for preexisting sRNAs (22–27). This may involve RdRP recruitment to transcripts with aberrant or unusual molecular features generated by other RNA polymerases (23, 24, 28, 29).

In the filamentous fungus *Neurospora crassa*, silencing, or “quelling,” of transgenic arrays relies on RdRP QDE-1, PIWI/PAZ protein QDE-2, DNA helicase QDE-3, and several other components, including the ssDNA-binding protein RPA (2). Interestingly, QDE-1 may trigger quelling by producing long aberrant ssRNA copies (aRNAs) of ssDNA intermediates that frequently arise in tandem-duplicated genomic sequences (30–32). This reaction depends on QDE-3 and RPA and is stimulated by DNA damage. QDE-1 can subsequently use its aRNA product as a template to produce dsRNA intermediates. These are subsequently converted into sRNAs that associate with QDE-2 and target complementary sequences. Thus, at least some RdRPs may initiate gene silencing *de novo*.

In line with their diverse biological functions, individual RdRPs and their protein complexes isolated from various sources show different enzymatic properties *in vitro*. These include polymerase-specific preferences between ssRNA *versus* ssDNA templates and primer-dependent *versus* primer-independent initiation of RNA synthesis (33–37). Some RdRPs can also function as template-independent terminal transferases (34, 35). Notably, two distinct template-dependent polymerization modes have been described for previously studied RdRPs: (*a*) processive synthesis of long double-stranded products, which is typically initiated at or close to the 3′-end of a single-stranded template using either primer-independent or so-called “back-priming” mechanisms, and (*b*) non-processive synthesis of sRNA copies initiated in a primer-independent manner at internal positions of a single-stranded template (33).

Different RdRPs appear to utilize the two modes with markedly different efficiencies. For example, Rdr1 from *Schizosaccharomyces pombe* or RDR6 from *Arabidopsis* efficiently synthesizes long products (34, 38), whereas RRF-1 from *Caenorhabditis elegans* specializes in production of sRNAs (39). These biochemical differences are consistent with *in vivo* evidence; long dsRNAs generated by Rdr1, RDR2, and RDR6 must be processed by Dicer/RNAse III-like endoribonucleases to generate functional small interfering RNAs (siRNAs), whereas sRNA products of RRF-1 apparently do not require further processing for their secondary siRNA function (16, 19, 20).

Purified QDE-1 from *N. crassa* can use both primer-independent and “back-priming” mechanisms *in vitro* (33, 35). The crystal structure of QDE-1 catalytic fragment, thus far the only known structure of a cell-encoded RdRP, suggests that this enzyme is a homodimer with the two subunits adopting either “closed” or “open” conformation (40). It has been proposed that the two structurally distinct conformations may help this remarkably versatile enzyme choose between different activities (40). However, in the absence of structural information for corresponding enzyme-substrate complexes, whether QDE-1 in fact remains a dimer upon template binding is unknown. Moreover, it remains to be seen whether other RdRPs can form homodimers, an important question, given that at least some RdRPs behave as monomers in solution (36).

On a more fundamental level, how new functional properties evolve in the RdRPs (and in other protein families for that matter) is poorly understood. Gene duplication followed by paralog divergence is a major driving force in protein evolution (41, 42), and it clearly contributed to RdRP diversification. Indeed, many species encode more than one distinct RdRP, with three paralogous genes present in *N. crassa* (*QDE-1*, *SAD-1*, and *RRP-3*), four in *C. elegans*, and six in *Arabidopsis* (14). The last eukaryotic common ancestor might have contained three functionally distinct RdRPs giving rise to the α, β, and γ branches of the RdRP genealogy, an arrangement that was further modified by lineage-specific gene duplications and losses (43).

Species-specific members of individual paralogous groups, referred to as orthologs, are typically assumed to have similar biological activities (44). However, it has been alternatively proposed that divergence of orthologous sequences might frequently result in acquisition of novel functional properties (45). Until recently, it has been difficult to investigate these possibilities experimentally because genomes of just a few distantly related model organisms have been sequenced completely. Here we took advantage of the increasing number of whole-genome sequences available for fungal species and examined evolutionary trends in QDE-1 orthologs using phylogenetic, biochemical, and structural approaches.

## Experimental Procedures

### 

#### 

##### Phylogenetic Analyses

Amino acid sequences of fungal polymerases were downloaded from OrthoDB (46) and aligned using MUSCLE (47). Phylogenetic trees were constructed in MEGA6 (48) by computing evolutionary distances using Poisson correction and inferring evolutionary history by the neighbor-joining method. Tree topology was tested using bootstrapping. Amino acid sequence conservation profiles were plotted in EMBOSS/plotcon using a 50-amino acid sliding window (49). Protein structures were color-coded according to interspecies conservation using Chimera (50). Chimera was also used to predict ligand positions in *N. crassa* QDE-1 apoenzyme (Protein Data Bank code 2J7N) (40) based on the known structure of the polymerase II elongation complex (Protein Data Bank code 1R9T) (51).

##### Protein Expression and Purification

Recombinant proteins were expressed and purified as described elsewhere (52, 53). Briefly, synthetic open reading frames (ORFs) encoding catalytic fragments of QDE-1*^Tte^* and QDE-1*^Mth^* were obtained from Genscript, and the sequence-encoding catalytic fragment of QDE-1*^Ncr^* (QDE-1ΔN) was amplified from pEM55 (33). Catalytically inactive QDE-1*^Mth^* D607A mutant with the DYDGD motif substituted with AYDGD was prepared by QuikChange mutagenesis (Agilent). The ORFs were amplified using primers shown in Table 1, cloned into the pFB-LIC-Bse (a gift from Opher Gileadi; Addgene plasmid 26108) using ligation-independent cloning (54), and subsequently transformed into DH10Bac (Life Technologies) to produce the recombinant bacmids. Viral stocks generated by introducing the bacmids into Sf9 insect cells were further amplified and used to infect Sf9 cells for large scale protein expression. Virus-infected cells were harvested by centrifugation at 4,000 × *g* for 15 min at 4 °C. Cell pellets were resuspended in 20 mm HEPES-NaOH, pH 8.0, 5 mm imidazole, 300 mm NaCl, 5% (v/v) glycerol with cOmplete EDTA-free protease inhibitors (Roche Applied Science) and subjected to sonication. Soluble fractions were isolated by centrifugation at 50,000 × *g* for 30 min at 4 °C and incubated with His tag purification resin (Roche Applied Science) for 1 h at room temperature. Non-specifically bound proteins were eluted by 20 mm HEPES-NaOH, pH 8.0, 15 mm imidazole, 300 mm NaCl, 5% (v/v) glycerol, and 0.5 mm tris(2-carboxyethyl)phosphine. His tag-containing proteins were eluted with 20 mm HEPES-NaOH, pH 8.0, 500 mm imidazole, 150 mm NaCl, 5% (v/v) glycerol, 0.5 mm tris(2-carboxyethyl)phosphine. Fractions containing the protein were concentrated using a 100 kDa cut-off concentrator (Sartorius) and further purified by size exclusion chromatography using Superdex 200 (GE Healthcare) pre-equilibrated with 20 mm HEPES-NaOH, pH 8.0, and 0.5 mm tris(2-carboxyethyl)phosphine additionally containing 150 mm NaCl and 5% (v/v) glycerol (QDE-1*^Tte^*), 300 mm NaCl and 5% (v/v) glycerol (QDE-1*^Ncr^*), or 500 mm NaCl and 10% (v/v) glycerol (QDE-1*^Mth^* and QDE-1*^Mth^* (D607A)). Eluted proteins were concentrated and stored at −80 °C. Chemicals were from Sigma-Aldrich unless stated otherwise.

**TABLE 1:** **Primers used in this study**

Name	Sequence (5′–3′)
QDE1_Ncr_forward	TACTTCCAATCCATGCTGGCTCGGAGCGAAGAAA
QDE1_Ncr_reverse	TATCCACCTTTACTGTCAATAATCGCCATTCCCTGTGAA
QDE1_Mth_forward	TACTTCCAATCCATGGTGATCCACTCCAGACTGC
QDE1_Mth_reverse	TATCCACCTTTACTGTCATTACTCGTCACCTGAATCGC
QDE1_Tte_forward	TACTTCCAATCCATGGAGGTGTACGCCCGCCTC
QDE1_Tte_reverse	TATCCACCTTTACTGTCAGTCCACGTCGTCACCGCG
D607A_forward	GACAAGTTGTCAGGTGGTGCCTACGATGGAG
D607A_reverse	GGCGAAGTCTCCATCGTAGGCACCACCTGAC

##### Protein Thermostability Assay

Protein thermostability was determined by monitoring temperature-induced fluorescence changes, as described elsewhere (55). Purified proteins were incubated at 1 mg/ml in the gel filtration buffer with 1,000-fold diluted SYPRO Orange stock (Life Technologies) in 96-well PCR plates (Bio-Rad) sealed with optical sealing tape (Bio-Rad). Fluorescence was measured using an iCycler iQ5 real-time PCR detection system (Bio-Rad) with excitation and emission wavelengths set at 490 and 575 nm, respectively. The temperature was increased from 20 to 90 °C with 1 °C increments, and the mixture was incubated for 12 s at each temperature. Protein melting temperatures were calculated using the iQ5 Optical System software, version 2.1 (Bio-Rad).

##### RNA Polymerase Assays

A synthetic single-stranded DNA (ssDNA) template (5′-CTGACTGCTTCCTGTTTCTGTTTTCTCTCCCCTCTTTTTCCTCATGTCCCACACCCCAACGGTCCCTTCATTTGTCTGTCTACCCTGTTGACAATTAATCATCGGCA-3′) was synthesized by Sigma. An ssRNA template corresponding to the s+ transcript of bacteriophage φ6 was produced from pLM659 (56) linearized with SmaI using the mMessage mMachine T7 transcription kit (Ambion). QDE-1 assays were carried out in 50 mm HEPES-NaOH, pH 7.8, 0.1 mm EDTA, 2% (v/v) Triton X-100, 100 mm NH_4_OAc, 2 mm MgCl_2_, 0.1 units/μl recombinant RNasin (Promega), 0.4 mm each of ATP, CTP, and GTP, 0.2 mm UTP, and 25 μCi/ml [α-^32^P]UTP (PerkinElmer Life Sciences). Reactions were initiated by adding a corresponding QDE-1 polymerase to a final concentration of 0.25 μg/μl followed by 1-h incubations at 30–65 °C. Reaction products were separated as described (33) using native or denaturing formaldehyde-containing agarose gel electrophoresis or urea-containing PAGE and analyzed by phosphorimaging (Typhoon Trio, GE Healthcare). An RNase protection assay was carried out by stopping the polymerase reactions with 250 mm NH_4_OAc and 10 mm EDTA followed by incubation with various concentrations of RNase ONE (Promega) for 1 h at 30 °C. The reaction products were analyzed by formaldehyde-containing agarose gel electrophoresis as described above. To quantify radioactivity incorporated into newly synthesized RNA products, reaction mixtures were spotted onto Whatman 3MM paper pretreated with 10% trichloroacetic acid (TCA) solution, washed in ice-cold 10% TCA solution for 10 min, rinsed twice with ice-cold 10% TCA solution and once with 95% ethanol, and allowed to air-dry. To determine the sum of incorporated and non-incorporated radioactivity, samples were spotted on Whatman 3MM paper and air-dried with no additional treatment. Radioactive signals were then analyzed by phosphorimaging.

##### Filter Binding Assay

Purified QDE-1*^Ncr^*, QDE-1*^Tte^*, or bovine serum albumin (BSA) control was incubated at 1 μm in the presence of binding buffer (50 mm HEPES-NaOH, pH 7.8, 0.1 mm EDTA, 2% (v/v) Triton X-100, 100 mm NH_4_OAc, and 2 mm MgCl_2_) and 0.03–10 μm 5′-CTTACTTGTATGGACATT-3′ ssDNA oligonucleotide labeled at the 5′-end using [γ-^32^P]ATP (PerkinElmer Life Sciences) and T4 polynucleotide kinase (New England Biolabs). Following a 10-min incubation at 20 °C, mixtures were passed through nitrocellulose membrane prewashed with the binding buffer using a Bio-Rad slot blot apparatus. The labeled RdRP-ssDNA complex retained on the membrane was quantified using phosphorimaging (Typhoon Trio, GE Healthcare).

##### Protein Crystallization, Diffraction Data Collection, and Structure Determination

Crystals of the QDE-1*^Tte^* catalytic fragment were obtained at 30 °C by mixing 2 μl of the protein solution at 2 mg/ml with 1 μl of crystallization solution (100 mm Tris-HCl, pH 8.0, 75 mm NaCl, 10% (w/v) PEG 10,000, 5 mm MgCl_2_, and 6 mm spermine). Crystals were transferred to a drop containing the crystallization solution supplemented with 10% (v/v) glycerol and incubated at 12 °C overnight. Before freezing, crystals were dehydrated at room temperature in two steps of 15 min each in the crystallization solution supplemented with 20 and 30% glycerol. X-ray diffraction data were collected to 3.19 Å resolution at 100 K at the PXIII beamline of the Swiss Light Source (Villingen, Switzerland) using a Pilatus 6M detector (Dectris). The crystal belonged to the P2_1_ space group with the following unit cell dimensions: *a* = 84.23 Å, *b* = 165.84 Å, *c* = 173.83 Å, and β = 90.10°. The data collection and structure refinement parameters are listed in Table 2. The structure was determined by molecular replacement using the known structure of the QDE-1*^Ncr^* catalytic fragment (Protein Data Bank code 2J7N) (40) as a search probe. The model was built interactively using Coot (57), and the structure was refined using REFMAC from the CCP4 package, with tight non-crystallographic symmetry restraints between the four independent monomers (each monomer was considered as a group) with individual atom isotropic temperature factors and TLS refinement (58). Each monomer of the QDE-1*^Tte^* fragment used for crystallization contains 1,034 residues, including the His tag and the tobacco etch virus protease cleavage site. Of these, 924/922 could be built in dimer A/B and 922/922 in dimer C/D in the two non-crystallographic dimers of the asymmetric unit (Table 2). Missing residues in the model belong to the N and C termini and flexible loops (Table 2). Because QDE-1*^Tte^* dimer A/B is better ordered than dimer C/D in the electron density map, we use it for subsequent comparisons.

**TABLE 2:** **Data collection and refinement statistics for QDE-Tth apoenzyme**

**Data collection statistics**	
Detector type	Pilatus 6M
Synchrotron	SLS PXIII
Wavelength (Å)	1.03320
Resolution (Å)	48.91–3.19 (3.38–3.19)*^a^*
Space group	P2_1_
Dimers per asymmetric unit	2
Unit cell parameters	
*a*, *b*, *c* (Å)	84.23, 165.84, 173.83
α, β, γ (degrees)	90, 90.10, 90
Measured reflections*^a^*	243,519 (38,635)
Unique reflections*^a^*	77,595 (12,236)
Redundancy	3.35 (1.6)
*I*/σ*^a^*	7.8 (3.0)
Completeness (%)*^a^*	98.6 (95.5)
*R*_merge_*^a^*	0.169 (0.399)

**Refinement statistics**	
Reflections used for refinement	75,503
Non-hydrogen atoms*^b^*	28,130
*R*_work_ (%)	21.30 (25.06)
*R*_free_ (%)	25.09 (26.20)
Root mean square deviations	
Bond lengths (Å)	0.015
Bond angles (degrees)	1.85
Mean *B*-factor (Å^2^)	
Protein overall (A, B, C, D)*^c^*	60.58 (16.32, 33.99, 64.8, 77.5)
Ramachandran plot	
Most favored (%)	83.2
Allowed (%)	13.8
Outliers (%)	1.4
Protein Data Bank code	5FSW

*^a^* The values for the highest resolution shell are shown in parenthesis.

*^b^* Residues missing from the model are as follows: 213–225, 566–567, 813–816, 867–875, 895–906, 923–925, 967–972, and 1007–1034.

*^c^* The *B*-factor values are given for all of the protein atoms and for each of the four polypeptide chains in the asymmetric unit.

##### Electron Microscopy (EM)

Purified QDE-1*^Tte^* was diluted to 10 μg/ml with 100 mm Tris-HCl, pH 7.5, 75 mm NaCl, 5 mm MgCl_2_, and 5% (v/v) glycerol. A volume of 4 μl of protein sample was applied to a glow-discharged carbon-coated transmission EM grid and stained with 2% (v/v) uranyl acetate. RdRP-ssDNA substrate complex was formed by incubating 1.3 μm QDE-1*^Tte^* with 1.7 μm (*i.e.* ∼10-fold higher than the apparent *K_d_* determined in the filter binding assay) DNA oligonucleotide 5′-CCTTAATTGTATAG-3′ before transferring acutely diluted mixture to a grid and subsequently staining with uranyl acetate as above. Images were recorded at a magnification of ×66,350 using a FEI T12 transmission electron microscope equipped with a 4K CCD camera (FEI) under low dose conditions. Single particles were selected and processed with the EMAN2 image-processing package (59). Initially, a total of 3,000 particles each of the QDE-1*^Tte^* apoenzyme and of QDE-1*^Tte^* preincubated with ssDNA were used for two-dimensional classifications and three-dimensional reconstructions. For both data sets, 10 initial models were generated with EMAN2 and compared with the 40 Å resolution filtered crystal structure of the QDE-1*^Tte^* dimer. The initial model structurally most similar to the crystal structure was selected for further refinement for both data sets to obtain a three-dimensional EM map at about 31 Å resolution without imposing any symmetry constraint. To obtain a higher resolution map of QDE-1*^Tte^* preincubated with ssDNA, a data set of 11,992 particles was used and processed as described above, which led to a “reference-free” three-dimensional EM map at a resolution of 20 Å. In parallel, the same data set for QDE-1*^Tte^* preincubated with ssDNA particles was processed as described above but with the crystal structure of apo-QDE-1*^Tte^* as a reference.

## Results

### 

#### 

##### Orthologs of N. crassa QDE-1 Belong to a Rapidly Evolving Protein Group

**FIGURE 1.**
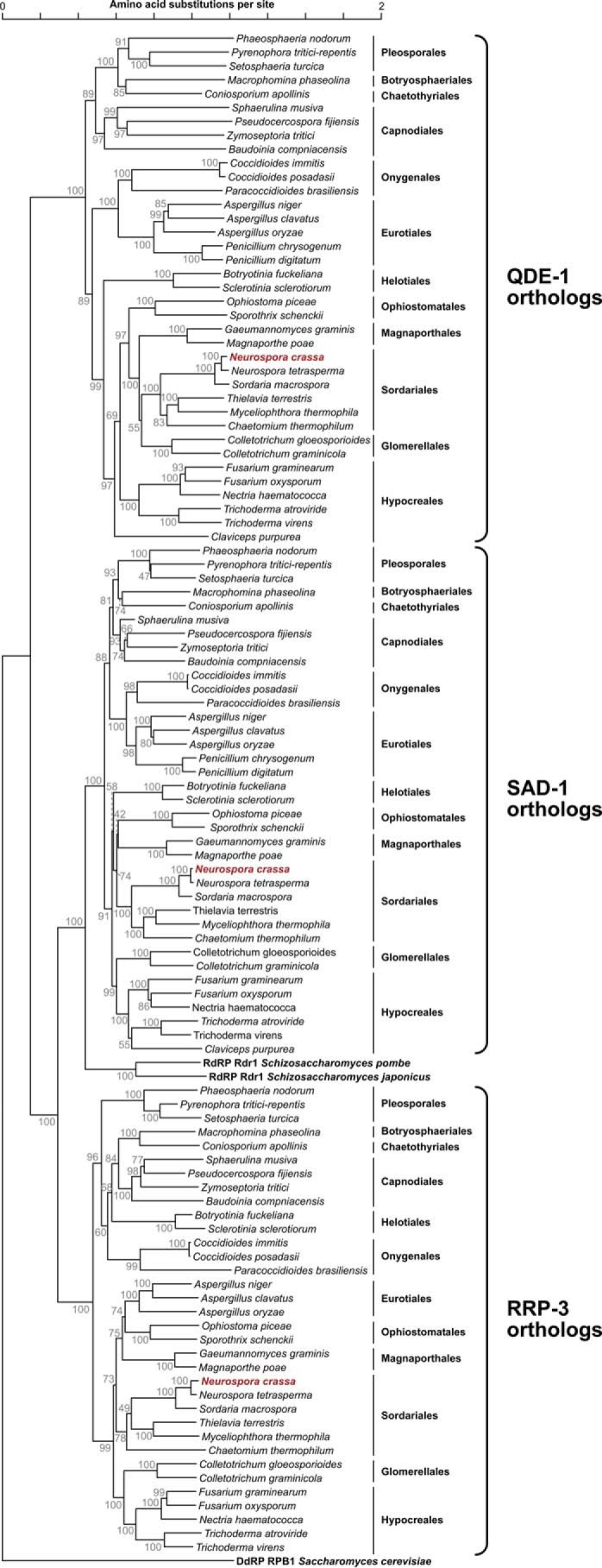
**Phylogenetic analysis of fungal QDE-1, SAD-1, and RRP-3 RdRPs.** QDE-1 and SAD-1 orthologs were detected in all 37 *Ascomycota* species used in this analysis, and RRP-3 was present in 34 species. The tree was constructed using the neighbor-joining method and drawn to scale with the evolutionary distances computed as numbers of amino acid substitutions per site. All positions with less than 50% site coverage were eliminated. Percentages of replicate trees in which the associated taxa clustered together in the bootstrap test (1,000 replicates) are shown beside the branches. *N. crassa* RdRPs are highlighted in *red*. The tree additionally shows *S. pombe* and *S. japonicus* fission yeast Rdr1 sequences (UniProt IDs RDR1_SCHPO and B6K409_SCHJY, respectively) clustering with the SAD-1 RdRP branch and the RPB1 subunit of the *S. cerevisiae* polymerase II DdRP used as an outgroup (UniProt ID RPB1_YEAST).

To gain insights into RdRP evolution, we analyzed corresponding genes from 40 taxonomically diverse fungi with completely sequenced genomes (Table 3 and Fig. 1). Most species encoded RdRP proteins clustering with *N. crassa* QDE-1, SAD-1, or RRP-3, except for three species from the Eurotiales and Hypocreales orders that had QDE-1 and SAD-1 but not RRP-3. *S. pombe* and *Schizosaccharomyces japonicus* had a single RdRP (Rdr1) related to SAD-1, and *Saccharomyces cerevisiae* had no RdRPs, as expected. Within each of the three groups, RdRPs clustered according to their taxonomic origin (Fig. 1). This topology suggested that the last common ancestor of fungi might have contained at least three RdRP paralogs that evolved as orthologously related lineages or were occasionally lost during speciation.

**TABLE 3:** **UniProt IDs of protein sequences used in phylogenetic analyses**

Order	Genus and species	QDE-1	SAD-1	RRP-3	RPB1	RPB2
Botryosphaeriales	*Macrophomina phaseolina*	K2RDJ0_MACPH	K2QNW5_MACPH	K2RK31_MACPH	K2R7Z7_MACPH	K2RU19_MACPH
Capnodiales	*Baudoinia compniacensis*	M2N109_BAUCO	M2NF26_BAUCO	M2LZJ3_BAUCO	M2N508_BAUCO	M2NI14_BAUCO
Capnodiales	*Pseudocercospora fijiensis*	M2Z6I8_MYCFI	M3BD53_MYCFI	M3AK73_MYCFI	M3B0A6_MYCFI	M3AZS4_MYCFI
Capnodiales	*Sphaerulina musiva*	M3B6Y6_SPHMS	M3CWK8_SPHMS	M3AUC5_SPHMS	M3C1A1_SPHMS	M3CKY8_SPHMS
Capnodiales	*Zymoseptoria tritici*	F9X3Z4_MYCGM	F9XQ35_MYCGM	F9XMI7_MYCGM	F9X8Y2_MYCGM	F9X8C0_MYCGM
Chaetothyriales	*Coniosporium apollinis*	R7YL14_CONA1	R7Z0Z6_CONA1	R7YZ34_CONA1	R7Z6D3_CONA1	R7Z672_CONA1
Eurotiales	*Aspergillus clavatus*	A1CAB8_ASPCL	A1CJE9_ASPCL	A1C791_ASPCL	A1CNZ4_ASPCL	A1CG49_ASPCL
Eurotiales	*Aspergillus niger*	G3Y0J3_ASPNA	G3YBG5_ASPNA	G3YEZ9_ASPNA	G3XRG6_ASPNA	G3XUX0_ASPNA
Eurotiales	*Aspergillus oryzae*	I8TQF9_ASPO3	I7ZKD2_ASPO3	I7ZNM0_ASPO3	I8TRP3_ASPO3	I8IG63_ASPO3
Eurotiales	*Penicillium chrysogenum*	B6HHR3_PENCW	B6HF55_PENCW	N/A	B6H9U6_PENCW	B6HM08_PENCW
Eurotiales	*Penicillium digitatum*	K9FWM5_PEND1	K9GG53_PEND1	N/A	K9FLF3_PEND1	K9FQU8_PEND1
Glomerellales	*Colletotrichum gloeosporioides*	L2FZH6_COLGN	L2G8J1_COLGN	L2GIQ1_COLGN	L2FJ99_COLGN	L2GHB3_COLGN
Glomerellales	*Colletotrichum graminicola*	E3QU45_COLGM	E3Q757_COLGM	E3QJU4_COLGM	E3Q4C8_COLGM	E3QBS9_COLGM
Helotiales	*Botryotinia fuckeliana*	M7U3A8_BOTF1	M7U8A4_BOTF1	M7U399_BOTF1	M7TRI5_BOTF1	M7UPJ1_BOTF1
Helotiales	*Sclerotinia sclerotiorum*	A7EDI7_SCLS1	A7F6D2_SCLS1	A7EX56_SCLS1	A7EKT1_SCLS	A7E6G0_SCLS1
Hypocreales	*Claviceps purpurea*	M1WAQ5_CLAP2	M1VU32_CLAP2	N/A	M1WH47_CLAP2	M1VVJ0_CLAP2
Hypocreales	*Fusarium graminearum*	I1RR02_GIBZE	I1RWN8_GIBZE	I1RD90_GIBZE	I1RBJ1_GIBZE	I1RG11_GIBZE
Hypocreales	*Fusarium oxysporum*	F9FKY2_FUSOF	F9FRB9_FUSOF	F9FSG6_FUSOF	F9FU19_FUSOF	F9F4K1_FUSOF
Hypocreales	*Nectria haematococca*	C7Z829_NECH7	C7YMG0_NECH7	C7YW47_NECH7	C7YI77_NECH7	C7ZA30_NECH7
Hypocreales	*Trichoderma atroviride*	G9P714_HYPAI	G9P3Q6_HYPAI	G9NRF8_HYPAI	G9P9P3_HYPAI	G9NY90_HYPAI
Hypocreales	*Trichoderma virens*	G9NC85_HYPVG	G9MQT5_HYPVG	G9NB73_HYPVG	G9MIB4_HYPVG	G9MQ39_HYPVG
Magnaporthales	*Gaeumannomyces graminis*	J3PGN9_GAGT3	J3PDJ5_GAGT3	J3P241_GAGT3	J3NK53_GAGT3	J3P8A5_GAGT3
Magnaporthales	*Magnaporthe poae*	M4G909_MAGP6	M4FQM6_MAGP6	M4G3Z1_MAGP6	M4G2S5_MAGP6	M4FRT3_MAGP6
Onygenales	*Coccidioides immitis*	J3GK9B6_COCIM	J3KGY1_COCIM	J2KMD3_COCIM	J3KB55_COCIM	J3K4V5_COCIM
Onygenales	*Coccidioides posadasii*	E9DBL8_COCPS	E9D4N0_COCPS	E9D8K1_COCPS	E9CY52_COCPS	E9DJ48_COCPS
Onygenales	*Paracoccidioides brasiliensis*	C1GJK3_PARBD	C1G807_PARBD	C1GG49_PARBD	C1GHL1_PARBD	C1GAQ4_PARBD
Ophiostomatales	*Ophiostoma piceae*	S3CKA4_OPHP1	S3BXM6_OPHP1	S3C5L6_OPHP1	S3CAX5_OPHP1	S3CNJ2_OPHP1
Ophiostomatales	*Sporothrix schenckii*	U7PQJ1_SPOS1	U7PX37_SPOS1	U7PKQ3_SPOS1	U7Q5K3_SPOS1	U7Q4W4_SPOS1
Pleosporales	*Phaeosphaeria nodorum*	Q0V1X8_PHANO	Q0V6X7_PHANO	Q0TXM0_PHANO	Q0U9K8_PHANO	Q0U9V8_PHANO
Pleosporales	*Pyrenophora tritici-repentis*	B2WKG5_PYRTR	B2WBK2_PYRTR	B2W8W4_PYRTR	B2W4S0_PYRTR	B2W4R0_PYRTR
Pleosporales	*Setosphaeria turcica*	R0IKE8_SETT2	R0JV11_SETT2	R0IYG1_SETT2	R0KQY5_SETT2	R0J2Z8_SETT2
Saccharomycetales	*Saccharomyces cerevisiae*	N/A	N/A	N/A	RPB1_YEAST	RPB2_YEAST
Schizosaccharomycetales	*Schizosaccharomyces pombe*	N/A	RDR1_SCHPO	N/A	RPB1_SCHPO	RPB2_SCHPO
Schizosaccharomycetales	*Schizosaccharomyces japonicus*	N/A	B6K409_SCHJY	N/A	B6JYP8_SCHJY	B6K5Q5_SCHJY
Sordariales	*Chaetomium thermophilum*	G0S3K7_CHATD	G0S1A8_CHATD	G0RYS7_CHATD	G0SCU7_CHATD	G0S9E0_CHATD
Sordariales	*Myceliophthora thermophila*	G2QGP0_THIHA	G2Q023_THIHA	G2QNN4_THIHA	G2Q1R2_THIHA	G2QG82_THIHA
Sordariales	*Neurospora crassa*	Q1K6C4_NEUCR	Q1K907_NEUCR	Q1K8P0_NEUCR	Q7SDN0_NEUCR	Q7RYF4_NEUCR
Sordariales	*Neurospora tetrasperma*	F8MKS5_NEUT8	F8MY77_NEUT8	F8MNF1_NEUT8	F8N2G6_NEUT8	F8MVQ3_NEUT8
Sordariales	*Sordaria macrospora*	F7VSI8_SORMK	F7VUX1_SORMK	F7VJY3_SORMK	F7VUQ0_SORMK	F7WAM6_SORMK
Sordariales	*Thielavia terrestris*	G2R911_THITE	G2R3N8_THITE	G2RGB9_THITE	G2RET7_THITE	G2QVH8_THITE

**FIGURE 2.**
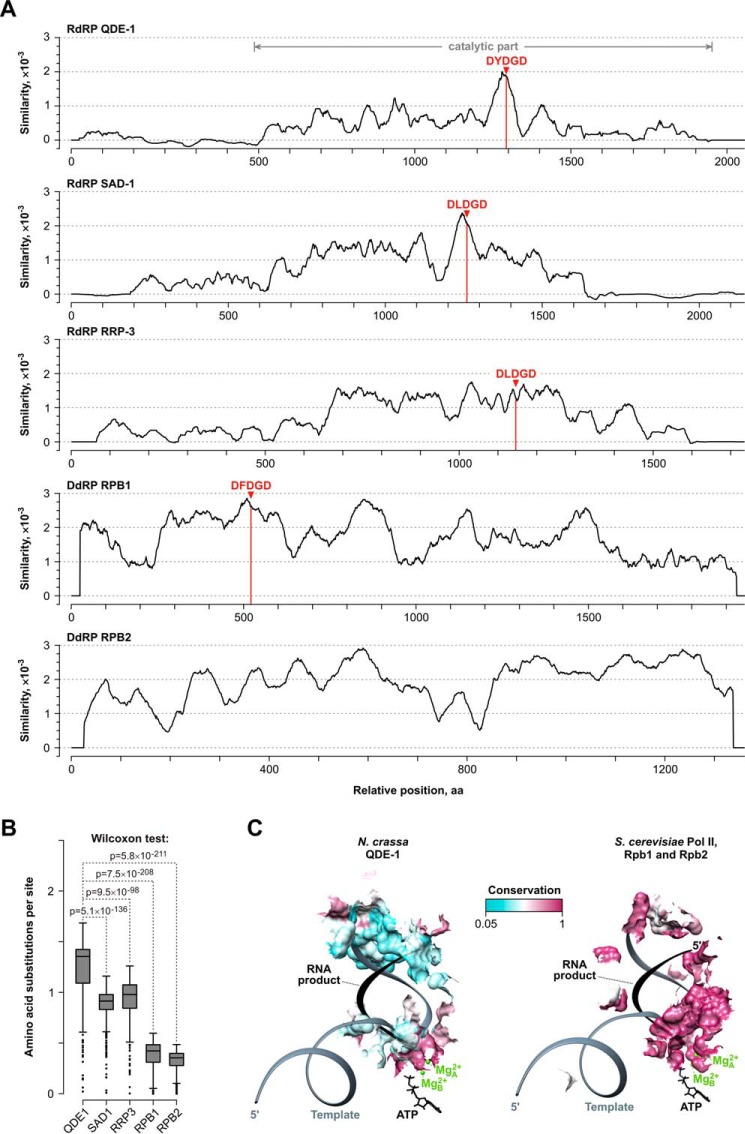
**QDE-1 orthologs rapidly diverge in evolution.** *A*, similarity plots for the QDE-1, SAD-1, and RRP-3 orthologs as well as for RPB1 and RPB2 (*bottom*) subunits of polymerase II DdRP from 37 fungal species containing more than one paralogous RdRP. *Red arrowheads* mark positions of the catalytic D(Y/F/L)DGD motif in the RdRP and RPB1 active centers (RPB2 lacks this sequence). The *gray dimension line* indicates the C-terminal part of QDE-1 previously shown to be enzymatically active (33). Note that the DYDGD-adjacent region in the QDE-1 orthologs is conserved noticeably better than the rest of the sequence. This contrasts with the rest of the proteins containing wider areas of relatively strong conservation. *B*, *box and whisker plot* comparison of amino acid substitution scores showing significantly higher divergence rate in QDE-1 proteins compared with their SAD-1, RRP-3, RPB1, and RPB2 counterparts. Corresponding Wilcoxon test *p* values are indicated at the *top. C*, surfaces proximal (≤4 Å) to the template (*dark gray*) and the nascent RNA product (*black*) in the “closed” subunit of *N. crassa* QDE-1 (QDE-1*^Ncr^*) (Protein Data Bank code 2J7N) (40) and *S. cerevisiae* polymerase II (Protein Data Bank code 1R9T) (51). Incoming ATP monomer and the two catalytic ions, Mg^2+^_A_ and Mg^2+^_B_, are shown in *black* and *green*, respectively. Protein surfaces are *colored* using *cyan* for low, *white* for intermediate, and *maroon* for high conservation. In the case of QDE-1*^Ncr^*, Mg^2+^_A_ position is determined experimentally, whereas template, RNA product, incoming ATP, and Mg^2+^_B_ are modeled based on the polymerase II elongation structure on the *right. Error bars* represent the standard errors.

Notably, the QDE-1 orthologs tended to be connected by longer branches than their SAD-1 and RRP-3 counterparts (Fig. 1). To identify regions accounting for their apparently accelerated evolution, we examined a QDE-1 conservation plot (Fig. 2). The non-catalytic N-terminal part showed extremely low conservation scores, as pointed out previously (33). However, even within the generally less divergent C-terminal part, a prominent peak of sequence conservation was detected only in the vicinity of the catalytic DYDGD motif (14, 33). This contrasted with the SAD-1 and RRP-3 plots that contained substantially broader regions of relatively high conservation. Conservation was even more uniform for two polymerase II subunits, RPB1 and RPB2, distantly related to cell-encoded RdRPs (40) (Fig. 2*A*). Quantitative analyses of amino acid substitution rates showed that QDE-1 was significantly more divergent than SAD-1, RRP-3, RPB1, and RPB2 (Fig. 2*B*).

**FIGURE 3.**
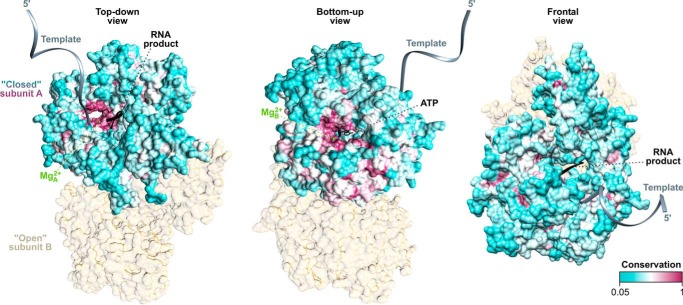
**Spatial clustering of evolutionarily conserved amino acid sequences in QDE-1Ncr structure.** *Surface representations* of the QDE-1*^Ncr^* protein homodimer show the “closed” A subunit *colored* according to interspecies conservation and the “open” B subunit *colored* in *beige*. The known position of Mg^2+^_A_ and predicted positions of the second catalytic Mg^2+^ ion (Mg^2+^_B_), an incoming ATP, a template, and an RNA product are also indicated. Note that highly conserved amino acid residues cluster in the vicinity of the catalytic center and the NTP pore.

Interestingly, conserved amino acid residues showed prominent clustering around the active center of the known crystal structure of the QDE-1*^Ncr^* apoenzyme interacting with one of the two catalytic Mg^2+^ ions (Mg^2+^_A_) (Protein Data Bank code 2J7N) (40) (Figs. 2*C* and 3). We modeled positions of other molecules participating in RNA polymerization, including an incoming ATP monomer, the second Mg^2+^ ion (Mg^2+^_B_), the template, and the RNA product based on the structure of the polymerase II elongation complex (Protein Data Bank code 1R9T) (51). This placed the ATP and Mg^2+^_B_ near the conserved surface of the nucleotide pore and the nascent 3′-end of the RNA product along with the corresponding template nucleotides near the conserved DYDGD loop and its Mg^2+^_A_ ligand (Figs. 2*C* and 3). On the other hand, a more distal segment of the template-product duplex egressing from the active center was surrounded by substantially more divergent QDE-1 surfaces (Figs. 2*C* and 3). Notably, all contacts made by the template and the nascent RNA with the RPB1 and RPB2 subunits of the polymerase II complex appear highly conserved in evolution (Fig. 2*C*). We concluded that the QDE-1 group is generally more divergent than its RdRP paralogs and DdRP relatives and that it shows unusually strong sequence variability outside of the active center and the NTP-interacting surfaces.

##### QDE-1 Orthologs Generate Markedly Different Combinations of Long and Short RNA Products

**FIGURE 4.**
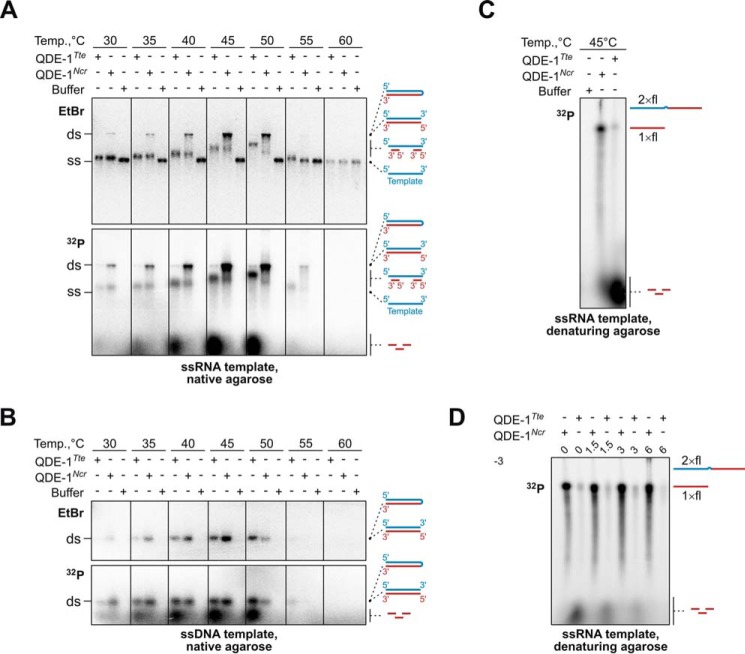
**QDE-1 orthologs have distinct enzymatic properties.** *A* and *B*, RNA polymerase activities of purified catalytic fragments of QDE-1*^Ncr^* and QDE-1*^Tte^* were assayed at 30–60 °C in the presence of an ssRNA (*A*) or an ssDNA template (*B*). The reaction products were separated by native agarose gel electrophoresis and visualized using ethidium bromide staining or ^32^P phosphorimaging, as indicated. Note that QDE-1*^Tte^* is substantially more efficient than QDE-1*^Ncr^* in generating sRNA products that migrate either at a low molecular weight position or in a template base-paired form. *C*, RNA polymerase activities of QDE-1*^Ncr^* and QDE-1*^Tte^* were assayed at 45 °C in the presence of a ssRNA template and analyzed by denaturing agarose gel electrophoresis. *D*, RNA products from *C* were incubated with increasing concentrations of RNase ONE or RNase ONE reaction buffer, as specified under “Experimental Procedures.” Positions of the 1× full template-length products of processive end-to-end polymerization initiated in a primer-independent manner and sRNA products of non-processive polymerization are indicated on the *right*. Also shown is an expected position of “back-primed” 2× template-length products, which QDE-1*^Ncr^* can generate for some but not all ssRNA templates (33).

**FIGURE 5.**
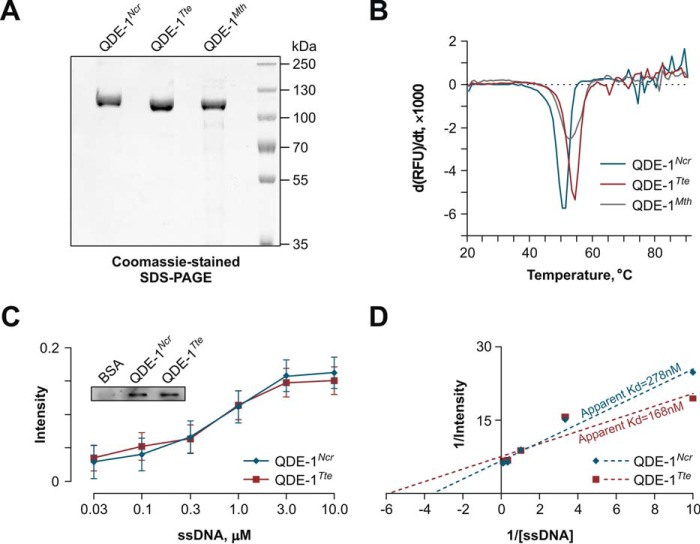
**Purification and biochemical characterization of QDE-1 orthologs.** *A*, SDS-PAGE analysis of purified recombinant QDE-1*^Ncr^*, QDE-1*^Tte^*, and QDE-1*^Mth^* catalytic fragments. *B*, thermostability analyses showing that the three proteins have comparable melting temperatures, 50.3, 54.0, and 52.3 °C, respectively. *C* and *D*, binding of a short ssDNA template by QDE-1*^Ncr^* and QDE-1*^Tte^* was analyzed using a slot blot filter-binding assay, as described under “Experimental Procedures.” Binding data in *C* are averaged from three independent experiments ± S.E. (*error bars*), and the *inset* shows an example of filter binding data for 1 μm ssDNA incubated with QDE-1*^Ncr^*, QDE-1*^Tte^*, or BSA control. *D*, a Lineweaver-Burk plot of the data in *C* used to determine apparent *K_d_* values for QDE-1*^Ncr^* and QDE-1*^Tte^*.

To examine whether divergent QDE-1 proteins had distinct functional properties, we purified catalytic fragments of QDE-1*^Ncr^* and its ortholog from *Thielavia terrestris* (QDE-1*^Tte^*) from the Chaetomiaceae family distantly related to *N. crassa* (Sordariaceae family) using a standardized protocol (see “Experimental Procedures”) and analyzed the RNA polymerase activity of these two proteins (Figs. 4 and 5). QDE-1*^Ncr^* is known to accept either ssRNA or ssDNA templates and generate their continuous end-to-end RNA copies through a primer-independent (*de novo*) or primer-dependent (“back-priming”) initiation mechanism (33, 35). A distinct primer-independent mode allows QDE-1*^Ncr^* to produce sRNA copies of internal template sequences (33). We therefore assayed QDE-1*^Ncr^* and QDE-1*^Tte^* RNA polymerase activities using recombinant ssRNA (2,948 nucleotides (nt)) and ssDNA (107 nt) templates and analyzed reaction products using native agarose gel electrophoresis. As expected (33), QDE-1*^Ncr^* readily synthesized detectable amounts of both long and short RNA copies migrating on native gels as full-length dsRNAs and partial dsRNA species, respectively (Fig. 4*A*).

Similar to its *N. crassa* ortholog, the newly analyzed QDE-1*^Tte^* was a highly active RNA polymerase (Fig. 4*A*). However, it showed a striking bias toward generating short RNA products rather than full-length copies (Fig. 4*A*). This activity produced readily detectable amounts of template-product duplexes migrating slower than the ssRNA template but faster than the corresponding full-length dsRNA on EtBr-stained gels (Fig. 4*A*). Gel autoradiography additionally revealed a prominent low molecular weight band probably corresponding to short RNA copies released from the template (Fig. 4*A*). A similarly migrating band of short RNAs was also a major reaction product in the ssDNA-programmed reactions containing QDE-1*^Tte^* but not QDE-1*^Ncr^* (Fig. 4*B*). On the other hand, both enzymes gave rise to full-length DNA-RNA template-product duplexes with comparable efficiencies (Fig. 4*B*).

To better understand the nature of RNA products, we analyzed ssRNA-programmed reactions by denaturing agarose gel electrophoresis (Fig. 4*C*). Under these conditions, short RNA copies quantitatively dissociated from the template and migrated at the expected low molecular weight position. This analysis additionally revealed RNA products of 1× template length, thus suggesting that, under conditions used in our RdRP assays, both QDE-1*^Ncr^* and QDE-1*^Tte^* can initiate end-to-end RNA synthesis in a predominantly primer-independent manner (33, 35) (Fig. 4*C*). Consistent with the native gel analyses, the ratio between short and long RNA products was noticeably higher for QDE-1*^Tte^* than for QDE-1*^Ncr^* (Fig. 4*C*). Importantly, the bias for short RNA products was consistently detected for several independently purified batches of QDE-1*^Tte^* and observed over a wide temperature range (Fig. 4, *A* and *B*). This ruled out the trivial explanation that the enzymatic differences between QDE-1*^Tte^* and QDE-1*^Ncr^* were related to a higher growth temperature limit of *T. terrestris* compared with *N. crassa* (60–62). In fact, the thermal stability of the QDE-1*^Tte^* protein exceeded that of QDE-1*^Ncr^* by only 3.7 °C (*T_m_* = 54.0 ± 0.0 °C *versus* T*_m_* = 50.3 ± 0.6 °C; Fig. 5*B*). The two polymerases also had comparable single-stranded template binding properties (Fig. 5, *C* and *D*). Thus, distinct QDE-1 orthologs may have markedly different RNA polymerization properties.

##### Evolutionary Divergence between QDE-1 Family Members Modulates Their Product Preferences

To ensure that functional differences between QDE-1*^Ncr^* and QDE-1*^Tte^* resulted from evolutionary innovation, we purified a catalytic fragment of QDE-1 from *Myceliophthora thermophila*, a Chaetomiaceae fungus more closely related to *T. terrestris* than to *N. crassa* (Figs. 1 and 5). The T*_m_* of QDE-1*^Mth^* was comparable with that of QDE-1*^Ncr^* and QDE-1*^Tte^* (T*_m_* = 52.3 ± 0.6 °C *versus* T*_m_* = 50.3 ± 0.6 °C and T*_m_* = 54.0 ± 0.0 °C, respectively; Fig. 5).

**FIGURE 6.**
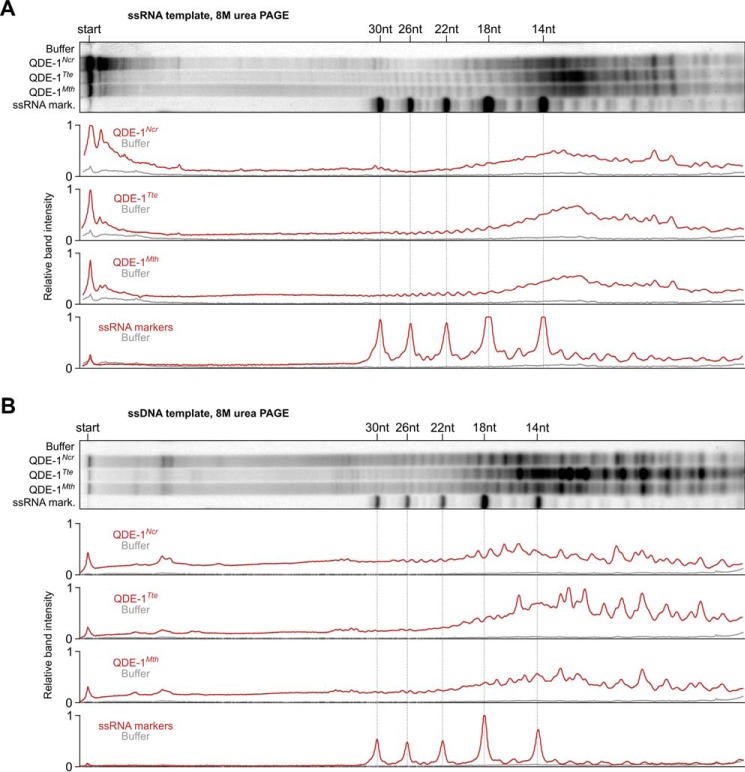
**QDE-1 orthologs synthesize different blends of sRNA products.** *A* and *B*, purified QDE-1*^Ncr^* and QDE-1*^Tte^* and QDE-1*^Mth^* were assayed at 45 °C with an ssRNA (*A*) or an ssDNA (*B*) template and analyzed by urea-containing 15% polyacrylamide gel electrophoresis. Note that in the presence of a long ssRNA, QDE-1*^Tte^* and QDE-1*^Mth^* generate a relatively higher fraction of sRNAs than QDE-1*^Ncr^*, especially those of 12 nt and 24–27 nt. On the other hand, QDE-1*^Ncr^* synthesizes a relatively larger amount of long RNA products migrating close to the *top* of the *lane*. Each of the three polymerases produces a unique combination of sRNA products from the ssDNA. In the lane scans provided at the *bottom* of each *panel*, maximal intensity of the 18-nt marker band is set to 1.

**FIGURE 7.**
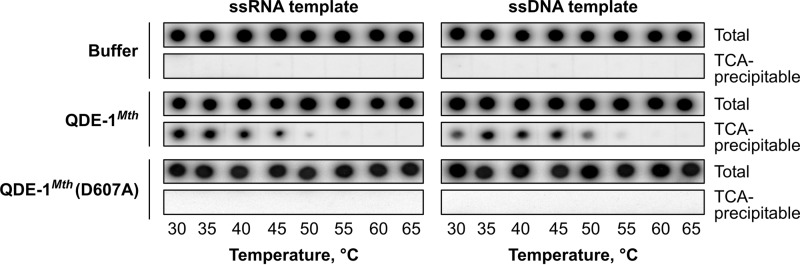
**Lack of RNA polymerase activity in the QDE-1Mth (D607A) mutant.** The QDE-1*^Mth^* (D607A) mutant containing the AYDGD sequence instead of the wild-type catalytic DYDGD motif shows no RNA polymerase activity in the presence of either ssRNA or ssDNA template and over a wide range of reaction temperatures.

To compare enzymatic properties of QDE-1*^Ncr^*, QDE-1*^Tte^*, and QDE-1*^Mth^*, we incubated the three polymerases with either ssRNA or ssDNA template for 1 h at 45 °C and separated the reaction products by urea-containing PAGE, affording simultaneous detection of long RNAs migrating at the top of the lane and sRNA products visualized at single-nucleotide resolution (Fig. 6). In ssRNA-programmed reactions, QDE-1*^Mth^* polymerase was clearly more efficient in producing short RNA products than QDE-1*^Ncr^*, albeit not to the same extent as QDE-1*^Tte^* (Fig. 6*A*). Moreover, QDE-1*^Ncr^* synthesized detectable amounts of ≤23-nt and ∼28–31-nt products but virtually no 24–27-nt-long sRNAs (Fig. 6*A*). On the other hand, both QDE-1*^Tte^* and QDE-1*^Mth^* efficiently produced 24–27-nt sRNA in addition to shorter products (Fig. 6*A*). Interestingly, each of the three polymerases generated a unique blend of sRNA products in ssDNA-programmed reactions (Fig. 6*B*). As an additional control, we purified a D607A mutant QDE-1*^Mth^*, where the first Asp residue of the DYDGD motif was mutated to Ala. As expected, the mutant protein lacked detectable nucleotidyltransferase activity (Fig. 7). We concluded that sequence divergence between QDE-1 orthologs appears to underlie differences in their activities.

##### QDE-1*^Tte^* Is Structurally Similar to QDE-1^Ncr^

**FIGURE 8.**
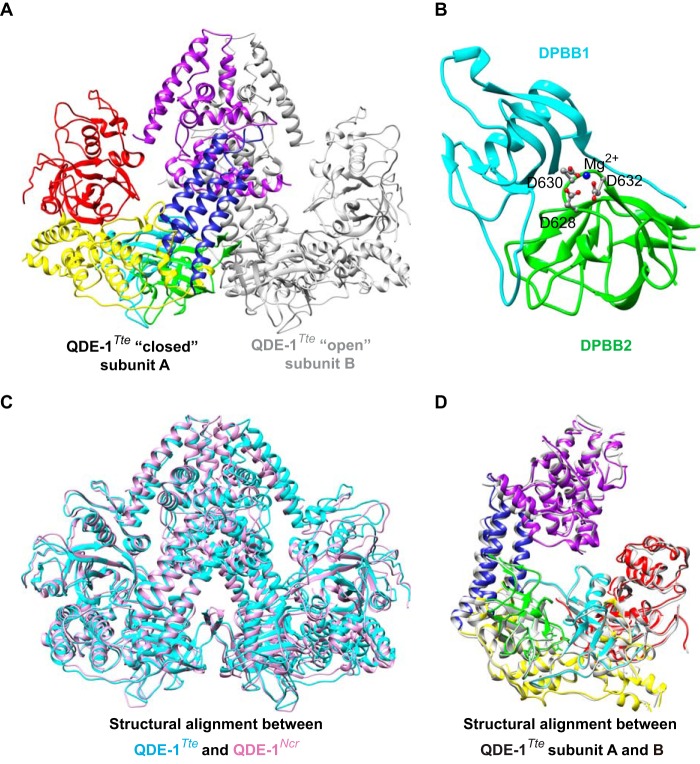
**QDE-1Tte crystal structure.** *A*, QDE-1*^Tte^* is an asymmetric dimer with each subunit containing a DPBB1 (shown in *cyan* for subunit A), a DPBB2 (*green*), a “head” (*magenta*), a “neck” (*blue*), and a “slab” domain (*red*). The entire B subunit is *colored* in *gray. B*, *magnified view* of the DPBB1 and DPBB2 domains with the three catalytic aspartate side chains shown as *balls* and *sticks. C*, structural alignment between QDE-1*^Tte^* and previously solved QDE-1*^Ncr^* structure showing a considerable overlap between the two proteins. *D*, structural alignment between subunits A and B QDE-1*^Tte^* demonstrating that subunit A adopts a more “closed” conformation than subunit B. The two subunits are *color-coded* as in *A*.

We next wondered whether distinct functional properties of QDE-1 orthologs might be due to major differences in spatial structures of these enzymes. This appeared plausible, given the degree of amino acid sequence divergence outside of the active center (see Fig. 2). To this end, we determined QDE-1*^Tte^* three-dimensional structure using x-ray crystallography. A 3.19 Å resolution trace of the QDE-1*^Tte^* polypeptide chain revealed two closely similar QDE-1*^Ncr^*-like homodimers in the asymmetric unit (root mean square deviation of 1.06 Å between dimer A/B and C/D for 917 α-carbon atoms). In each dimer, the two monomers are related by a non-crystallographic dyad (Fig. 8*A*).

Other structural details of QDE-1*^Tte^* also resembled those of QDE-1*^Ncr^* (40). For example, the QDE-1*^Tte^* dimer had a pyramidal shape with a pair of double-ψ β-barrel domains (DPBB1 and DPBB2; residues 312–413 and 535–639) located at the base of each subunit (Fig. 8, *A* and *B*). The “head” (residues 457–507 and 822–1006 of the catalytic fragment) and the “neck” domains (residues 428–532 and 784–821) of the two subunits adopted a slightly tilted back-to-back position (Fig. 8*A*), and the “slab” domain (residues 1–244) of each subunit protruded from the catalytic domain toward the corresponding head domain. The five domains formed an extensive groove, probably accommodating the template and the nascent RNA product, with the catalytic site located at the bottom (Fig. 8, *A* and *B*). In general, Cα atoms in the QDE-1*^Ncr^* and QDE-1*^Tte^* dimers superimposed very well with a root mean square deviation score of 1.34 Å for 917 α-carbon atoms (Fig. 8*C*).

As found previously for QDE-1*^Ncr^*, one of the two QDE-1*^Tte^* subunits adopted a slightly more closed conformation than the other. Superposition of 922 Cα atoms between the two monomers (dimer A/B) using the SSM server returned a root mean square deviation of 2.15 Å (Fig. 8*D*). The largest conformational differences between the two monomers occurred closer to the C-terminal end of the polypeptide chain. The homodimer interface is stabilized by a total of 119 and 115 residues of monomer A and B, respectively (63). Most residues at the interface originate from the upper “neck” and “head” domains, where the subunits display the largest structural differences (Fig. 8*C*). These data suggest that QDE-1*^Ncr^* and QDE-1*^Tte^* have remarkably similar tertiary and quaternary structures despite their primary sequence differences.

##### QDE-1^Tte^ Can Function as a Dimer in Solution

**FIGURE 9.**
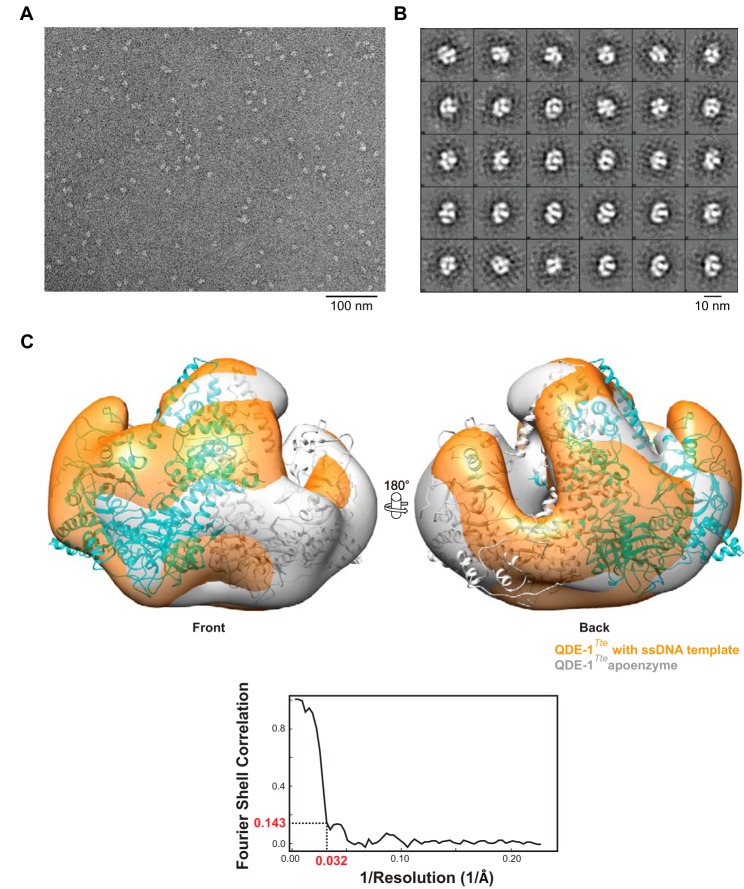
**Electron microscopy reconstructions of the apo- and ssDNA-bound QDE-1Tte structures.** *A*, a total of 3,000 particles were used for the analysis presented in this figure. A negatively stained electron micrograph shows particles of QDE-1*^Tte^* dimers in complex with an ssDNA template. *B*, representative reference-free two-dimensional class averages of the particles in *A. C*, overlay of QDE-1*^Tte^* structures determined by reference-free EM reconstruction in the presence (*orange*) or absence of a ssDNA template (*gray*). The crystal structure of the unbound QDE-1*^Tte^* dimer is overlaid, with its subunits *colored* in *cyan* and *gray*, respectively. Note that at this resolution of about 31 Å (1/0.032 Å^−1^ shown by the Fourier shell correlation map as an *inset*, both the apoenzyme and the ssDNA complex appear as structurally similar dimers.

**FIGURE 10.**
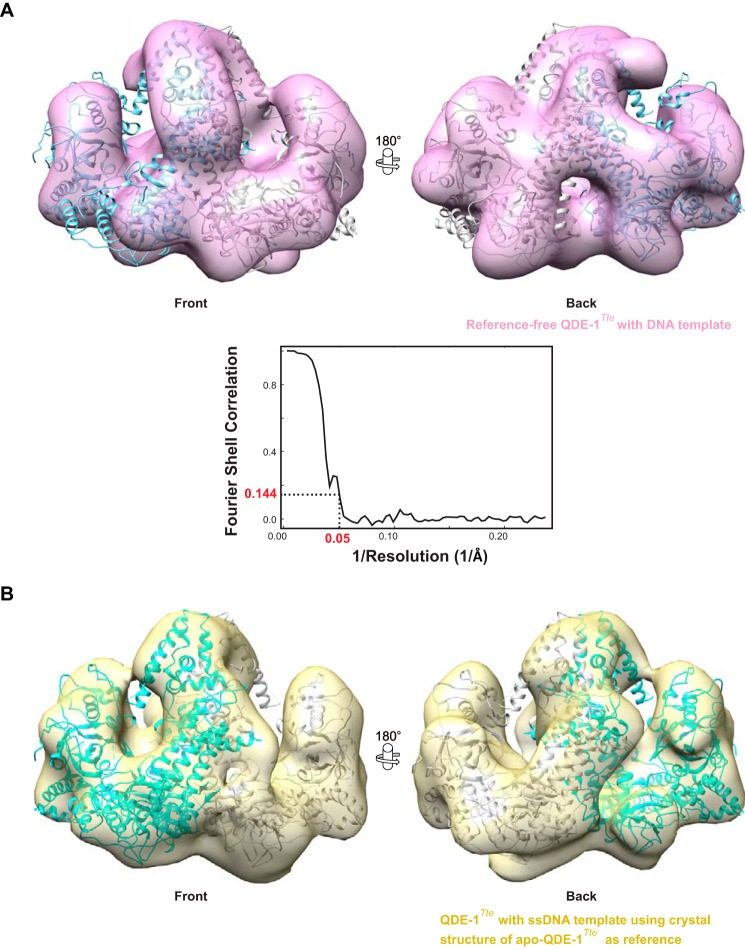
**Higher resolution EM structures of the QDE-1Tte ssDNA complex.** EM data analysis using a total of 11,992 particles is shown for the reference-free QDE-1*^Tte^* (*A*) or using the crystal structure of unbound QDE-1*^Tte^* as a reference (*B*). The crystal structure of QDE-1*^Tte^* was fitted into each of the EM reconstructions. For each reconstruction, a *front* and *back view* is provided with subunits of QDE-1*^Tte^* rendered in *cyan* and *gray. A* additionally shows the Fourier shell correlation curve from the final refined density map of QDE-1*^Tte^*-ssDNA. In both reconstructions, the resolution is ∼20 Å (1/0.051 Å^−1^).

Extensive contacts between the A and B subunits observed in the QDE-1*^Ncr^* and QDE-1*^Tte^* crystals suggested that these proteins may form catalytically active dimers in solution with the two monomers oscillating between the closed and open conformations. To test this possibility directly, we used EM to compare negatively stained images of the QDE-1*^Tte^* apoenzyme and its complex with a ssDNA template (Fig. 9). using reference-free two-dimensional class averaging (Fig. 9*B*). These initial EM reconstructions carried out using 3,000 apoenzyme and 3,000 ssDNA bound particles revealed largely similar pyramid-shaped homodimer structures, each containing two quasi-symmetric grooves and showing at this resolution a generally good fit with the QDE-1*^Tte^* crystal structure obtained without bound DNA (Fig. 9*C*). As in the crystal structure, one of the two template-product grooves in the QDE-1*^Tte^* dimer adopted a more open conformation in the EM reconstructions (Fig. 9*C*). To obtain a better resolved solution structure of QDE-1*^Tte^* in the presence of ssDNA, we extended our EM data analysis to include a total of 11,992 particles. This allowed us to visualize this complex to a resolution of 20 Å (Fig. 10, *A* and *B*). As expected, the structural fit between the EM and crystal structures of QDE-1*^Tte^* further improved when the EM map was prepared using the crystal structure as a reference (Fig. 10*B*). Of note, even this improved resolution was not sufficiently high to unambiguously assign density for the ssDNA. Likewise, understanding of the subtle conformational changes in the QDE-1*^Tte^* dimer upon template binding will require a higher resolution structure of the RdRP-ssDNA complex, a challenge that will be addressed in the future. Nonetheless, our present data strongly suggest that QDE-1 orthologs function as dimers in solution.

## Discussion

This study suggests that fungal QDE-1 orthologs underwent an unusually rapid sequence divergence outside of the catalytic center and the nucleotide-binding site. This corresponds to a significantly higher amino acid substitution rate in this group compared with its RdRP paralogs and distant DdRP relatives (Fig. 2). Consistent with their stronger divergence from QDE-1*^Ncr^* than from each other (Fig. 1), QDE-1*^Tte^* and QDE-1*^Mth^* differ from QDE-1*^Ncr^* in their enhanced ability to generate sRNA copies and reduced ability to synthesize long RNA products (Fig. 4). Because QDE-1*^Ncr^*, QDE-1*^Tte^*, and QDE-1*^Mth^* originate from a single taxonomic order (Sordariales), these data argue that protein functions can undergo substantial changes over relatively short periods of evolutionary history. Notably, this effect was not due to a recent gene duplication event followed by functional specialization of the newly emerged paralogs because our BLAST searches did not reveal any additional RdRP gene in the *N. crassa*, *T. terrestris*, and *M. thermophile* genomes besides *QDE-1*, *SAD-1*, and *RRP-3*.

What could be the biological significance of such accelerated functional evolution? One possible answer relates to the role of RNA silencing in cellular defense against viruses and transposable elements (3, 5, 6). Different species encounter distinct sets of pathogens, which necessitate corresponding changes in cellular defense mechanisms (64). Interestingly, *N. crassa* is a mesophilic fungus colonizing burnt vegetation and occasionally living plants (60, 61), whereas *T. terrestris* and *M. thermophila* typically inhabit self-heating composts (62). Exposure to diverse environments and correspondingly different sets of biohazards might have exerted substantial evolutionary pressure on cellular immunity factors, including QDE-1. An interesting direction for future studies would be to test whether this also rewired RNA silencing pathways in a more fundamental manner (*e.g.* incorporating sRNA products of QDE-1*^Tte^* and QDE-1*^Mth^* into signal amplification loops similar to the secondary siRNA pathway in *C. elegans* (16, 19–21, 39)).

The striking structural similarity between QDE-1*^Tte^* and QDE-1*^Ncr^* uncovered in this work (Fig. 8) suggests that evolution in this group probably proceeded through alteration of functionally important surface residues rather than major changes in the protein fold. In other words, QDE-1 orthologs show a combination of structural robustness and functional innovability (65). Although further work will be required to identify specific structural elements underlying functional divergence in the QDE-1 group, these will probably include amino acid residues directly interacting with the template-product duplex or affecting protein flexibility. Indeed, structural alignment of QDE-1*^Ncr^* or QDE-1*^Tte^* with the yeast polymerase II elongation complex (Protein Data Bank code 1R9T) (51) using structural similarity between the QDE-1 DPBB2 domain and the only DPBB domain of the polymerase II RPB1 subunit suggests that the QDE-1 enzyme may have to undergo major conformational changes to allow egress of template-product duplexes longer than ∼10 base pairs.

The EM reconstructions presented here provide an unprecedented insight into solution structure of an RdRP enzyme (Figs. 9 and 10). In line with the earlier prediction (40), QDE-1 assembles into a pyramid-shaped homodimer with each of the two subunits containing a groove suitable for template-product binding. One of the two subunits in the EM density maps adopts a more “closed” conformation than the other. This supports the “two-stroke motor” model of QDE-1 activity that was proposed earlier based on the x-ray structure of QDE-1*^Ncr^* apoenzyme (40). Importantly, our data indicate that QDE-1 retains its dimeric form in the presence of a single-stranded DNA template (Figs. 9 and 10).

We finally note that the efficient synthesis of sRNA products by the newly isolated QDE-1 enzymes might facilitate a range of research and diagnostic applications. QDE-1*^Tte^* appears to generate more than one copy of a given template sequence (Fig. 4, *A* and *B*). Therefore, this enzyme might be especially useful for amplifying either an entire nucleic acid target or its parts accessible to the polymerase. Combined with deep sequencing technology, this may open up new possibilities in high-throughput analyses of the transcriptome composition, RNA conformation and ribonucleoprotein complex structure. Enzymatic properties of QDE-1*^Tte^* could be further improved by knowledge-based mutagenesis of its evolutionarily variable parts or by “shuffling” corresponding sequences with their counterparts from other QDE-1 orthologs.

In conclusion, our study argues that acquisition of novel enzymatic properties through divergence of orthologous sequences could be a more common evolutionary scenario than anticipated previously. This work also improves our understanding of molecular mechanisms underlying RdRP functions and expands the existing molecular biology toolkit. We predict that further comparative analyses of this remarkably diverse class of enzymes will be a rewarding experience for evolutionary biologists and biochemists alike.

## Author Contributions

X. Q. and D. A. D. expressed the proteins (QDE-1*^Ncr^*, QDE-1*^Mth^*, QDE-1*^Tte^*, and QDE-1*^Mth^* (D607A)). X. Q. purified the proteins, conducted the thermofluor assay, crystallized QDE-1*^Tte^*, and solved its structure. X. Q. and F. M. H. conducted the polymerase activity assay. Y. H. W. generated the mutated polymerase QDE-1*^Mth^* (D607A). S. B. conducted the EM and refined the EM structure of QDE-1*^Tte^*. F. M. H. and E. V. M. conducted phylogenetic analysis. J. L. collected the diffraction data, with A. E. S., and refined the crystal structure of QDE-1*^Tte^*. X. Q., E. V. M., and J. L. wrote the paper.

## Footnotes

4 The abbreviations used are:

sRNA: small RNA
RdRP: RNA-dependent RNA polymerases
aRNA: aberrant ssRNA
nt: nucleotide(s)
DPBB: double-ψ β-barrel.

## References

[1] Bologna N. G., and Voinnet O. (2014) The diversity, biogenesis, and activities of endogenous silencing small RNAs in Arabidopsis. Annu. Rev. Plant Biol. 65, 473–503 24579988 10.1146/annurev-arplant-050213-035728

[2] Chang S. S., Zhang Z., and Liu Y. (2012) RNA interference pathways in fungi: mechanisms and functions. Annu. Rev. Microbiol. 66, 305–323 22746336 10.1146/annurev-micro-092611-150138 PMC4617789

[3] Zhou R., and Rana T. M. (2013) RNA-based mechanisms regulating host-virus interactions. Immunol. Rev. 253, 97–111 23550641 10.1111/imr.12053 PMC3695692

[4] Holoch D., and Moazed D. (2015) RNA-mediated epigenetic regulation of gene expression. Nat. Rev. Genet. 16, 71–84 25554358 10.1038/nrg3863 PMC4376354

[5] Szittya G., and Burgyán J. (2013) RNA interference-mediated intrinsic antiviral immunity in plants. Curr. Top. Microbiol. Immunol. 371, 153–181 23686235 10.1007/978-3-642-37765-5_6

[6] Nayak A., Tassetto M., Kunitomi M., and Andino R. (2013) RNA interference-mediated intrinsic antiviral immunity in invertebrates. Curr. Top. Microbiol. Immunol. 371, 183–200 23686236 10.1007/978-3-642-37765-5_7

[7] Dumesic P. A., and Madhani H. D. (2014) Recognizing the enemy within: licensing RNA-guided genome defense. Trends Biochem. Sci. 39, 25–34 24280023 10.1016/j.tibs.2013.10.003 PMC3902128

[8] Calo S., Shertz-Wall C., Lee S. C., Bastidas R. J., Nicolás F. E., Granek J. A., Mieczkowski P., Torres-Martínez S., Ruiz-Vázquez R. M., Cardenas M. E., and Heitman J. (2014) Antifungal drug resistance evoked via RNAi-dependent epimutations. Nature 513, 555–558 25079329 10.1038/nature13575 PMC4177005

[9] Ghildiyal M., and Zamore P. D. (2009) Small silencing RNAs: an expanding universe. Nat. Rev. Genet. 10, 94–108 19148191 10.1038/nrg2504 PMC2724769

[10] Kim V. N., Han J., and Siomi M. C. (2009) Biogenesis of small RNAs in animals. Nat. Rev. Mol. Cell Biol. 10, 126–139 19165215 10.1038/nrm2632

[11] Ipsaro J. J., and Joshua-Tor L. (2015) From guide to target: molecular insights into eukaryotic RNA-interference machinery. Nat. Struct. Mol. Biol. 22, 20–28 25565029 10.1038/nsmb.2931 PMC4450863

[12] Wilson R. C., and Doudna J. A. (2013) Molecular mechanisms of RNA interference. Annu. Rev. Biophys. 42, 217–239 23654304 10.1146/annurev-biophys-083012-130404 PMC5895182

[13] Burroughs A. M., Ando Y., and Aravind L. (2014) New perspectives on the diversification of the RNA interference system: insights from comparative genomics and small RNA sequencing. Wiley Interdiscip. Rev. RNA 5, 141–181 24311560 10.1002/wrna.1210 PMC4066877

[14] Wassenegger M., and Krczal G. (2006) Nomenclature and functions of RNA-directed RNA polymerases. Trends Plant Sci. 11, 142–151 16473542 10.1016/j.tplants.2006.01.003

[15] Cogoni C., and Macino G. (1999) Gene silencing in *Neurospora crassa* requires a protein homologous to RNA-dependent RNA polymerase. Nature 399, 166–169 10335848 10.1038/20215

[16] Baulcombe D. C. (2007) Molecular biology: amplified silencing. Science 315, 199–200 17218517 10.1126/science.1138030

[17] Fei Q., Xia R., and Meyers B. C. (2013) Phased, secondary, small interfering RNAs in posttranscriptional regulatory networks. Plant Cell 25, 2400–2415 23881411 10.1105/tpc.113.114652 PMC3753373

[18] Axtell M. J., Jan C., Rajagopalan R., and Bartel D. P. (2006) A two-hit trigger for siRNA biogenesis in plants. Cell 127, 565–577 17081978 10.1016/j.cell.2006.09.032

[19] Pak J., and Fire A. (2007) Distinct populations of primary and secondary effectors during RNAi in *C. elegans*. Science 315, 241–244 17124291 10.1126/science.1132839

[20] Sijen T., Steiner F. A., Thijssen K. L., and Plasterk R. H. (2007) Secondary siRNAs result from unprimed RNA synthesis and form a distinct class. Science 315, 244–247 17158288 10.1126/science.1136699

[21] Bagijn M. P., Goldstein L. D., Sapetschnig A., Weick E. M., Bouasker S., Lehrbach N. J., Simard M. J., and Miska E. A. (2012) Function, targets, and evolution of *Caenorhabditis elegans* piRNAs. Science 337, 574–578 22700655 10.1126/science.1220952 PMC3951736

[22] Gent J. I., Lamm A. T., Pavelec D. M., Maniar J. M., Parameswaran P., Tao L., Kennedy S., and Fire A. Z. (2010) Distinct phases of siRNA synthesis in an endogenous RNAi pathway in *C. elegans* soma. Mol. Cell 37, 679–689 20116306 10.1016/j.molcel.2010.01.012 PMC2838994

[23] Carradec Q., Götz U., Arnaiz O., Pouch J., Simon M., Meyer E., and Marker S. (2015) Primary and secondary siRNA synthesis triggered by RNAs from food bacteria in the ciliate Paramecium tetraurelia. Nucleic Acids Res. 43, 1818–1833 25593325 10.1093/nar/gku1331 PMC4330347

[24] Dumesic P. A., Natarajan P., Chen C., Drinnenberg I. A., Schiller B. J., Thompson J., Moresco J. J., Yates J. R. 3rd, Bartel D. P., and Madhani H. D. (2013) Stalled spliceosomes are a signal for RNAi-mediated genome defense. Cell 152, 957–968 23415457 10.1016/j.cell.2013.01.046 PMC3645481

[25] Calo S., Nicolás F. E., Vila A., Torres-Martínez S., and Ruiz-Vázquez R. M. (2012) Two distinct RNA-dependent RNA polymerases are required for initiation and amplification of RNA silencing in the basal fungus *Mucor circinelloides*. Mol. Microbiol. 83, 379–394 22141923 10.1111/j.1365-2958.2011.07939.x

[26] Haag J. R., Ream T. S., Marasco M., Nicora C. D., Norbeck A. D., Pasa-Tolic L., and Pikaard C. S. (2012) *In vitro* transcription activities of Pol IV, Pol V, and RDR2 reveal coupling of Pol IV and RDR2 for dsRNA synthesis in plant RNA silencing. Mol. Cell 48, 811–818 23142082 10.1016/j.molcel.2012.09.027 PMC3532817

[27] Lee S. R., and Collins K. (2007) Physical and functional coupling of RNA-dependent RNA polymerase and Dicer in the biogenesis of endogenous siRNAs. Nat. Struct. Mol. Biol. 14, 604–610 17603500 10.1038/nsmb1262

[28] Luo Z., and Chen Z. (2007) Improperly terminated, unpolyadenylated mRNA of sense transgenes is targeted by RDR6-mediated RNA silencing in *Arabidopsis*. Plant Cell 19, 943–958 17384170 10.1105/tpc.106.045724 PMC1867362

[29] Gazzani S., Lawrenson T., Woodward C., Headon D., and Sablowski R. (2004) A link between mRNA turnover and RNA interference in *Arabidopsis*. Science 306, 1046–1048 15528448 10.1126/science.1101092

[30] Lee H. C., Chang S. S., Choudhary S., Aalto A. P., Maiti M., Bamford D. H., and Liu Y. (2009) qiRNA is a new type of small interfering RNA induced by DNA damage. Nature 459, 274–277 19444217 10.1038/nature08041 PMC2859615

[31] Lee H. C., Aalto A. P., Yang Q., Chang S. S., Huang G., Fisher D., Cha J., Poranen M. M., Bamford D. H., and Liu Y. (2010) The DNA/RNA-dependent RNA polymerase QDE-1 generates aberrant RNA and dsRNA for RNAi in a process requiring replication protein A and a DNA helicase. PLoS Biol. 10.1371/journal.pbio.1000496 PMC2950127 20957187

[32] Yang Q., Ye Q. A., and Liu Y. (2015) Mechanism of siRNA production from repetitive DNA. Genes Dev. 29, 526–537 25691092 10.1101/gad.255828.114 PMC4358405

[33] Makeyev E. V., and Bamford D. H. (2002) Cellular RNA-dependent RNA polymerase involved in posttranscriptional gene silencing has two distinct activity modes. Mol. Cell 10, 1417–1427 12504016 10.1016/s1097-2765(02)00780-3

[34] Curaba J., and Chen X. (2008) Biochemical activities of *Arabidopsis* RNA-dependent RNA polymerase 6. J. Biol. Chem. 283, 3059–3066 18063577 10.1074/jbc.M708983200 PMC2629599

[35] Aalto A. P., Poranen M. M., Grimes J. M., Stuart D. I., and Bamford D. H. (2010) *In vitro* activities of the multifunctional RNA silencing polymerase QDE-1 of *Neurospora crassa*. J. Biol. Chem. 285, 29367–29374 20647305 10.1074/jbc.M110.139121 PMC2937969

[36] Talsky K. B., and Collins K. (2010) Initiation by a eukaryotic RNA-dependent RNA polymerase requires looping of the template end and is influenced by the template-tailing activity of an associated uridyltransferase. J. Biol. Chem. 285, 27614–27623 20622019 10.1074/jbc.M110.142273 PMC2934629

[37] Devert A., Fabre N., Floris M., Canard B., Robaglia C., and Crété P. (2015) Primer-dependent and primer-independent initiation of double stranded RNA synthesis by purified *Arabidopsis* RNA-dependent RNA polymerases RDR2 and RDR6. PLoS One 10, e0120100 25793874 10.1371/journal.pone.0120100 PMC4368572

[38] Motamedi M. R., Verdel A., Colmenares S. U., Gerber S. A., Gygi S. P., and Moazed D. (2004) Two RNAi complexes, RITS and RDRC, physically interact and localize to noncoding centromeric RNAs. Cell 119, 789–802 15607976 10.1016/j.cell.2004.11.034

[39] Aoki K., Moriguchi H., Yoshioka T., Okawa K., and Tabara H. (2007) *In vitro* analyses of the production and activity of secondary small interfering RNAs in *C. elegans*. EMBO J. 26, 5007–5019 18007599 10.1038/sj.emboj.7601910 PMC2140100

[40] Salgado P. S., Koivunen M. R., Makeyev E. V., Bamford D. H., Stuart D. I., and Grimes J. M. (2006) The structure of an RNAi polymerase links RNA silencing and transcription. PLoS Biol. 4, e434 17147473 10.1371/journal.pbio.0040434 PMC1750930

[41] Long M., VanKuren N. W., Chen S., and Vibranovski M. D. (2013) New gene evolution: little did we know. Annu. Rev. Genet. 47, 307–333 24050177 10.1146/annurev-genet-111212-133301 PMC4281893

[42] Chen S., Krinsky B. H., and Long M. (2013) New genes as drivers of phenotypic evolution. Nat. Rev. Genet. 14, 645–660 23949544 10.1038/nrg3521 PMC4236023

[43] Zong J., Yao X., Yin J., Zhang D., and Ma H. (2009) Evolution of the RNA-dependent RNA polymerase (RdRP) genes: duplications and possible losses before and after the divergence of major eukaryotic groups. Gene 447, 29–39 19616606 10.1016/j.gene.2009.07.004

[44] Koonin E. V. (2005) Orthologs, paralogs, and evolutionary genomics. Annu. Rev. Genet. 39, 309–338 16285863 10.1146/annurev.genet.39.073003.114725

[45] Studer R. A., and Robinson-Rechavi M. (2009) How confident can we be that orthologs are similar, but paralogs differ? Trends Genet. 25, 210–216 19368988 10.1016/j.tig.2009.03.004

[46] Kriventseva E. V., Tegenfeldt F., Petty T. J., Waterhouse R. M., Simão F. A., Pozdnyakov I. A., Ioannidis P., and Zdobnov E. M. (2015) OrthoDB v8: update of the hierarchical catalog of orthologs and the underlying free software. Nucleic Acids Res. 43, D250–D256 25428351 10.1093/nar/gku1220 PMC4383991

[47] Edgar R. C. (2004) MUSCLE: multiple sequence alignment with high accuracy and high throughput. Nucleic Acids Res. 32, 1792–1797 15034147 10.1093/nar/gkh340 PMC390337

[48] Tamura K., Stecher G., Peterson D., Filipski A., and Kumar S. (2013) MEGA6: Molecular Evolutionary Genetics Analysis version 6.0. Mol. Biol. Evol. 30, 2725–2729 24132122 10.1093/molbev/mst197 PMC3840312

[49] Rice P., Longden I., and Bleasby A. (2000) EMBOSS: the European Molecular Biology Open Software Suite. Trends Genet. 16, 276–277 10827456 10.1016/s0168-9525(00)02024-2

[50] Pettersen E. F., Goddard T. D., Huang C. C., Couch G. S., Greenblatt D. M., Meng E. C., and Ferrin T. E. (2004) UCSF Chimera: a visualization system for exploratory research and analysis. J. Comput. Chem. 25, 1605–1612 15264254 10.1002/jcc.20084

[51] Westover K. D., Bushnell D. A., and Kornberg R. D. (2004) Structural basis of transcription: nucleotide selection by rotation in the RNA polymerase II active center. Cell 119, 481–489 15537538 10.1016/j.cell.2004.10.016

[52] Shrestha B., Smee C., and Gileadi O. (2008) Baculovirus expression vector system: an emerging host for high-throughput eukaryotic protein expression. Methods Mol. Biol. 439, 269–289 18370110 10.1007/978-1-59745-188-8_19

[53] Structural Genomics Consortium, China Structural Genomics Consortium, Northeast Structural Genomics Consortium, Gräslund S., Nordlund P., Weigelt J., Hallberg B. M., Bray J., Gileadi O., Knapp S., Oppermann U., Arrowsmith C., Hui R., Ming J., dhe-Paganon S., et al (2008) Protein production and purification. Nat. Methods 5, 135–146 18235434 10.1038/nmeth.f.202 PMC3178102

[54] Savitsky P., Bray J., Cooper C. D., Marsden B. D., Mahajan P., Burgess-Brown N. A., and Gileadi O. (2010) High-throughput production of human proteins for crystallization: the SGC experience. J. Struct. Biol. 172, 3–13 20541610 10.1016/j.jsb.2010.06.008 PMC2938586

[55] Santos S. P., Bandeiras T. M., Pinto A. F., Teixeira M., Carrondo M. A., and Romão C. V. (2012) Thermofluor-based optimization strategy for the stabilization and crystallization of *Campylobacter jejuni* desulforubrerythrin. Protein Expr. Purif. 81, 193–200 22051151 10.1016/j.pep.2011.10.001

[56] Gottlieb P., Strassman J., Qiao X., Frilander M., Frucht A., and Mindich L. (1992) *In vitro* packaging and replication of individual genomic segments of bacteriophage φ 6 RNA. J. Virol. 66, 2611–2616 1560520 10.1128/jvi.66.5.2611-2616.1992 PMC241014

[57] Emsley P., and Cowtan K. (2004) Coot: model-building tools for molecular graphics. Acta Crystallogr. D Biol. Crystallogr. 60, 2126–2132 15572765 10.1107/S0907444904019158

[58] Collaborative Computational Project, Number 4 (1994) The CCP4 suite: programs for protein crystallography. Acta Crystallogr. D 50, 760–763 15299374 10.1107/S0907444994003112

[59] Tang G., Peng L., Baldwin P. R., Mann D. S., Jiang W., Rees I., and Ludtke S. J. (2007) EMAN2: an extensible image processing suite for electron microscopy. J. Struct. Biol. 157, 38–46 16859925 10.1016/j.jsb.2006.05.009

[60] Turner B. C., Perkins D. D., and Fairfield A. (2001) Neurospora from natural populations: a global study. Fungal Genet. Biol. 32, 67–92 11352529 10.1006/fgbi.2001.1247

[61] Kuo H. C., Hui S., Choi J., Asiegbu F. O., Valkonen J. P., and Lee Y. H. (2014) Secret lifestyles of *Neurospora crassa*. Sci. Rep. 4, 5135 24875794 10.1038/srep05135 PMC4038807

[62] Berka R. M., Grigoriev I. V., Otillar R., Salamov A., Grimwood J., Reid I., Ishmael N., John T., Darmond C., Moisan M. C., Henrissat B., Coutinho P. M., Lombard V., Natvig D. O., Lindquist E., et al (2011) Comparative genomic analysis of the thermophilic biomass-degrading fungi *Myceliophthora thermophila* and *Thielavia terrestris*. Nat. Biotechnol. 29, 922–927 21964414 10.1038/nbt.1976

[63] Krissinel E., and Henrick K. (2007) Inference of macromolecular assemblies from crystalline state. J. Mol. Biol. 372, 774–797 17681537 10.1016/j.jmb.2007.05.022

[64] Obbard D. J., Gordon K. H., Buck A. H., and Jiggins F. M. (2009) The evolution of RNAi as a defence against viruses and transposable elements. Philos. Trans. R. Soc. Lond. B Biol. Sci. 364, 99–115 18926973 10.1098/rstb.2008.0168 PMC2592633

[65] Tóth-Petróczy A., and Tawfik D. S. (2014) The robustness and innovability of protein folds. Curr. Opin. Struct. Biol. 26, 131–138 25038399 10.1016/j.sbi.2014.06.007

